# Comparative expression of Toll-like receptors and inflammatory cytokines in pigs infected with different virulent porcine reproductive and respiratory syndrome virus isolates

**DOI:** 10.1186/1743-422X-10-135

**Published:** 2013-04-30

**Authors:** Lili Zhang, Jie Liu, Juan Bai, Xiaoye Wang, Yufeng Li, Ping Jiang

**Affiliations:** 1 Key Laboratory of Animal Diseases Diagnostic and Immunology, Ministry of Agriculture, College of Veterinary Medicine, Nanjing Agricultural University, Nanjing 210095, China

**Keywords:** PRRSV, Immunogenicity, Pathogenicity, TLRs, Cytokines

## Abstract

**Background:**

Porcine reproductive and respiratory syndrome virus (PRRSV) is largely responsible for heavy economic losses in the swine industry worldwide because of its high mutation rate and subsequent emergence of virulent strains. However, the immunological and pathological responses of pigs to PRRSV strains with different virulence have not been completely elucidated.

**Methods:**

Twenty-four piglets were divided into 4 groups (n = 6 each) and inoculated with highly pathogenic PRRSV isolate BB0907 (HP), low pathogenic PRRSV NT0801 (LP), LP derivative strain NT0801-F70 (LP-der), and DMEM medium (control), respectively. The changes in TLR2, 3, 7, and 8 gene expression and TNF-α, IL-1β, IL-6, IFN-γ, and IL-10 secretion were evaluated using real-time PCR and ELISA at 6, 9, and 15 days post inoculation (d.p.i.). The cytokine levels were evaluated in the supernatants of porcine alveolar macrophages (PAMs) and peripheral blood mononuclear cells (PBMCs) following stimulation with LTA, poly(I:C), CL097, and PRRSV individually.

**Results:**

HP caused more severe clinical signs and pathological lesions in swine than LP and LP-der had almost no virulence compared with LP. The serum levels of IL-1β, IL-6, TNF-α, and IFN-γ were increased in HP-infected piglets, which were greater than in those infected with LP or LP-der. The mRNA levels of TLR3, 7, and 8 were significantly up-regulated in PAMs in HP-infected pigs compared to those in groups LP and LP-der. Furthermore, TNF-α and IL-1β secretion in PAMs from group LP was statistically greater than those from the control group after stimulation with either poly(I:C) or CL097. Meanwhile, TNF-α, IL-1β, and IL-6 levels in CL097-stimulated PBMCs from HP-infected pigs were markedly higher than those from the LP- and LP-der-infected groups.

**Conclusions:**

We found that HP was a stronger inducer of TLR 3, 7, and 8 expression and IL-1β, IL-6, TNF-α, and IFN-γ production compared to LP and LP-der. HP enhanced production of TNF-α, IL-1β, and IL-6 in PBMCs following CL097-stimulation more than LP and LP-der, whereas LP enhanced the secretion of TNF-α and IL-1β in poly(I:C)- and CL097-stimulated PAMs. Our data regarding cellular reactivity to different isolates should be useful in the development of more efficacious vaccines.

## Background

Porcine reproductive and respiratory syndrome virus (PRRSV) is an enveloped positive-sense single-stranded RNA virus of the family *Arteriviridae*[1], which induces abortion in pregnant sows and gilts, and causes respiratory distress with increased susceptibility to secondary infections in piglets and growing pigs [2,3]. In China, highly pathogenic PRRSV (HP-PRRSV) has been isolated and identified as the causative agent of a large-scale PRRSV outbreak that has been on-going since 2006 and is characterized by a genomic marker with a 30-amino acid (aa) deletion in the nonstructural protein 2 (NSP2)-coding region, compared to that in the PRRSV VR-2332 strain [4-6]. However, the pathological responses of different virulent PRRSV strains remain unknown.

Viral infections initiate a series of cellular events that lead to the generation of an antiviral state both in the infected cells and surrounding tissues [7]. Toll-like receptors (TLRs) are key components of the host innate recognition system, in which each TLR family detects distinct microbial pathogen-associated molecular patterns (PAMPs) and triggers the activation of specific signaling pathways, thereby inducing the transcription of inflammatory and/or anti-inflammatory cytokines [8]. For example, TLR3 recognizes double-stranded (ds) viral and synthetic RNA, such as polyriboinosinic polyribocytidylic acid (poly(I:C)) [9]. Once engaged, TLR3 triggers the activation of interferon regulatory factor 3 (IRF-3), a transcription factor with a critical role in the induction of type I interferon (INF) and nuclear factor kappa-light-chain-enhancer of activated B cells (NF-κB) through signaling processes that require the protein toll-interleukin-1 receptor domain-containing adaptor inducing IFN-β (TRIF) [10,11]. Single-stranded (ss) RNA is the ligand recognized by TLR7 and 8. These TLRs also trigger IRF-7-mediated type I IFN production upon activation, but unlike TLR3, the induction of IFN by TLR7 and 8 is coupled to the adaptor protein myeloid differentiation primary response gene 88 and not to TRIF. This recognition initiates signaling cascades that result in transcription factor activation ultimately leading to production of pro-inflammatory cytokines, chemokines, and/or anti-viral cytokines [12].

PRRSV is difficult to control due to its high mutation rate and the emergence of virulent strains [13-15], which may differently modulate immune responses based on cytokine expression [13-15] and PRRSV distribution in swine [13-15]. In the present study, three pathogenic PRRSV strains (highly pathogenic, HP; low pathogenic, LP; and low-derivative, LP-der) were analyzed and the modulation of TLR2, TLR3, TLR7, and TLR8 gene expression and tumor necrosis factor alpha (TNF-α), interleukin (IL)-1β, IL-6, IFN-γ, and IL-10 secretion were observed in vivo and in vitro. Our results showed that TLR3, TLR7, and TLR8 expression was correlated to PRRSV virulence, which is consistent with cytokine levels in response to different infectious agents.

## Results

### Clinical signs and body temperature changes

**Figure 1:**
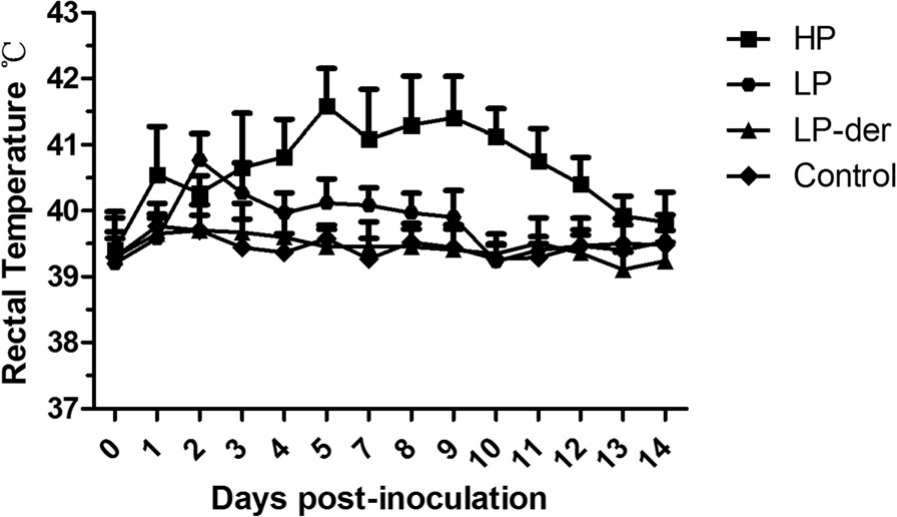
**Rectal temperature changes in pigs at 0 to 15 days post-inoculation of highly pathogenic PRRSV strain BB0907 (HP), low pathogenic PRRSV strain NT0801 (LP), NT0801 derivative strain NT0801-F70 (LP-der; propagated in MARC-145 cells to 70 passages), and Dulbecco’s modified Eagle’s medium (DMEM) (Control).** Each data point represents the mean ± standard deviation (SD) generated from 6 pigs in each group.

After PRRSV challenge, all pigs infected with the HP PRRSV SY0608 strain had high fever (≥ 41.0°C) and displayed a range of clinical signs, including inappetence, lethargy, rough hair coats, dyspnea, periocular edema, coughing, wheezing, and anhelation. Some pigs also showed eyelid edema, blue ears (cyanosis), muscle tremors, and diarrhea. Moreover, two of six pigs died at 9 days post-inoculation (d.p.i.), while pigs in the low pathogenic PRRSV NT0801 strain (LP) group showed a slight fluctuation in rectal temperatures and similar minor clinical signs at 15 d.p.i. Pigs in two groups, the NT0801 derivative strain NT0801-F70 (propagated in cultured African green monkey kidney-derived (MARC-145) cells to 70 passages) (group LP-der) and Dulbecco’s modified Eagle’s medium (DMEM) (as the control group), had no clinical signs or significant increases in body temperature (Figure 1). The scores of clinical signs in the HP group were significantly higher than those in the other groups (*p* < 0.05) (Table 1).

**Table 1:** **The clinical sign scores of the pigs after challenge and their lung lesions recorded at 15 d.p.i.**^**a**^

**Groups**	**Clinical signs scores (± SD)**^**b**^	**Lung lesions scores (± SD)**^**c**^
HP	10.4 ±1.22A	57.5 ± 5.34A
LP	7.2 ±0.92A	45.2 ± 9.23A
LP-der	1.8 ±0.35B	5.3 ± 4.82B
Control	0	0

^a^Within each column, values followed by different letters (A, B) are significantly different (*p* < 0.05).

^b^Clinical sign scores are the sum of daily observations of behavior, respiration, and cough according to the severity of the illness.

^c^Evaluation of the percentage of the entire lung affected by pneumonia.

### PRRSV Viremia

**Figure 2:**
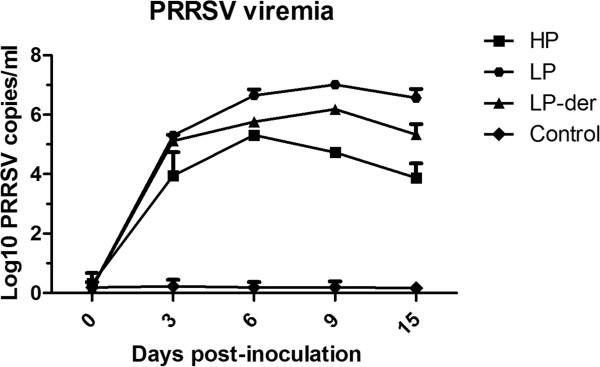
**PRRSV viremia in pigs at 0 to 15 days post-inoculation with HP, LP, and LP-der PRRSV strains and DMEM medium (Control).** Each data point represents the mean ± SD of the Log10 PRRSV cDNA copies/mL, as determined by quantitative PCR from the peripheral blood samples of 6 pigs in each group.

At 0, 3, 6, 9, and 15 d.p.i., pig blood samples were collected and serum PRRSV content was determined. As shown in Figure 2, pigs in group LP produced the highest levels of PRRSV viremia, which was significantly higher than those in groups LP-der and HP at 9 and 15 d.p.i. (*p* < 0.05) (Figure 2), while no viral content was detected in serum from the control pigs.

### Pathological examination

At 15 d.p.i., all pigs were euthanized for pathological examinations, which showed that all members of the HP-PRRSV group had diffuse tan consolidation of the lungs and occasional enlargement of the lymph nodes and spleen. As shown in Table 1, the lung lesion scores of HP-PRRSV-inoculated pigs were significantly higher than those in the other groups (*p* < 0.05), while mild lung lesions were observed in group LP, which were more severe than those in group LP-der.

**Figure 3:**
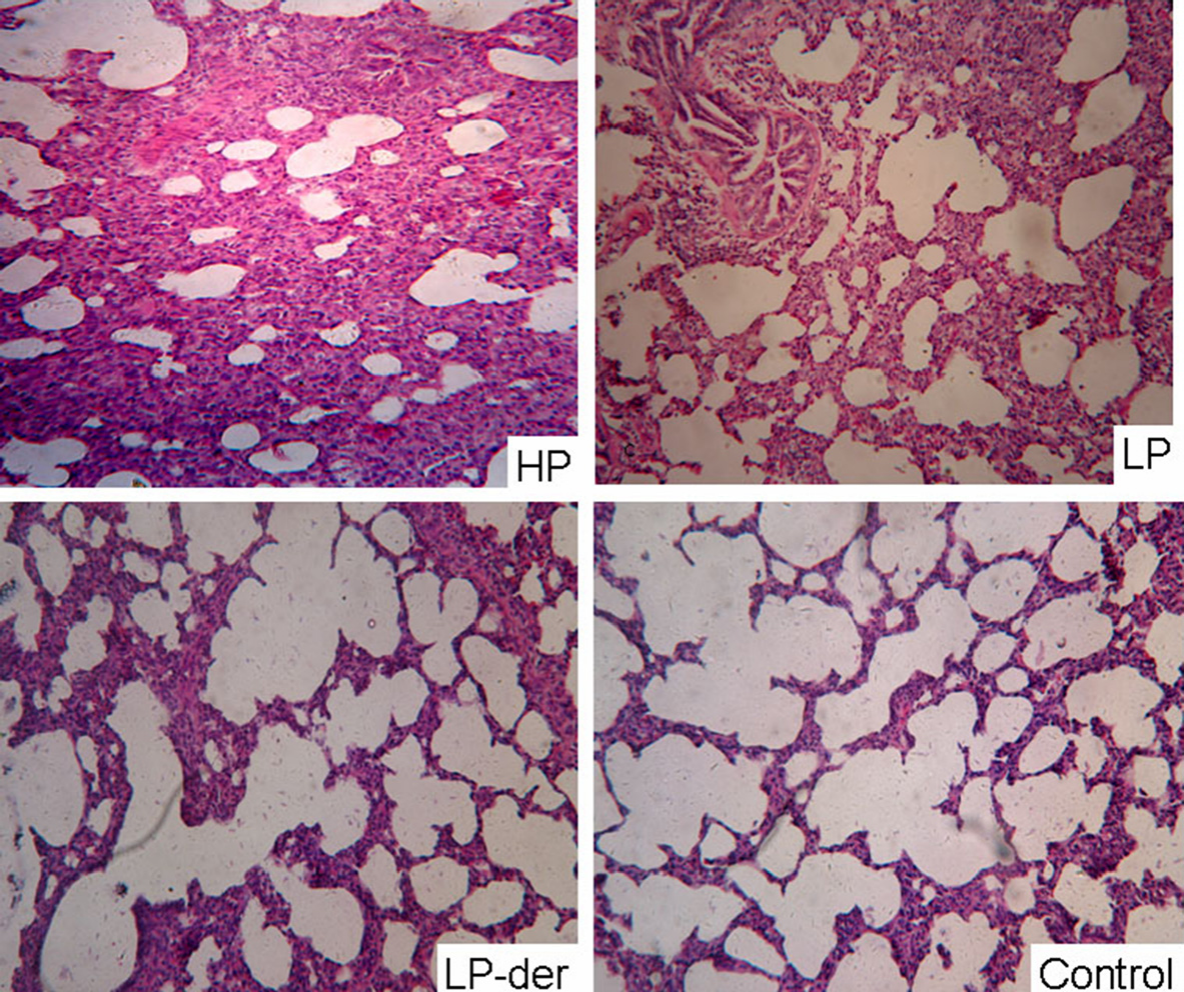
**Pathological examination of lung tissues of pigs from groups HP, LP, LP-der PRRSV and the control group at 15 d.p.i.** The tissues were stained with hematoxylin and eosin (magnification, x200).

On histological examination, the lung lesions in group HP-PRRSV pigs were characterized by thickened alveolar septa, infiltration with intensive lymphomononuclear cells, and hyperplasia of the bronchiolar epithelium (Figure 3A), whereas those in group LP-PRRSV exhibited mild interstitial pneumonia, which was lighter than that in group HP-PRRSV. The group LP-der pigs only exhibited light interstitial pneumonia (Figure 3), while there were no pathological changes in lung tissues from the control pigs.

### Serum cytokine levels

**Figure 4:**
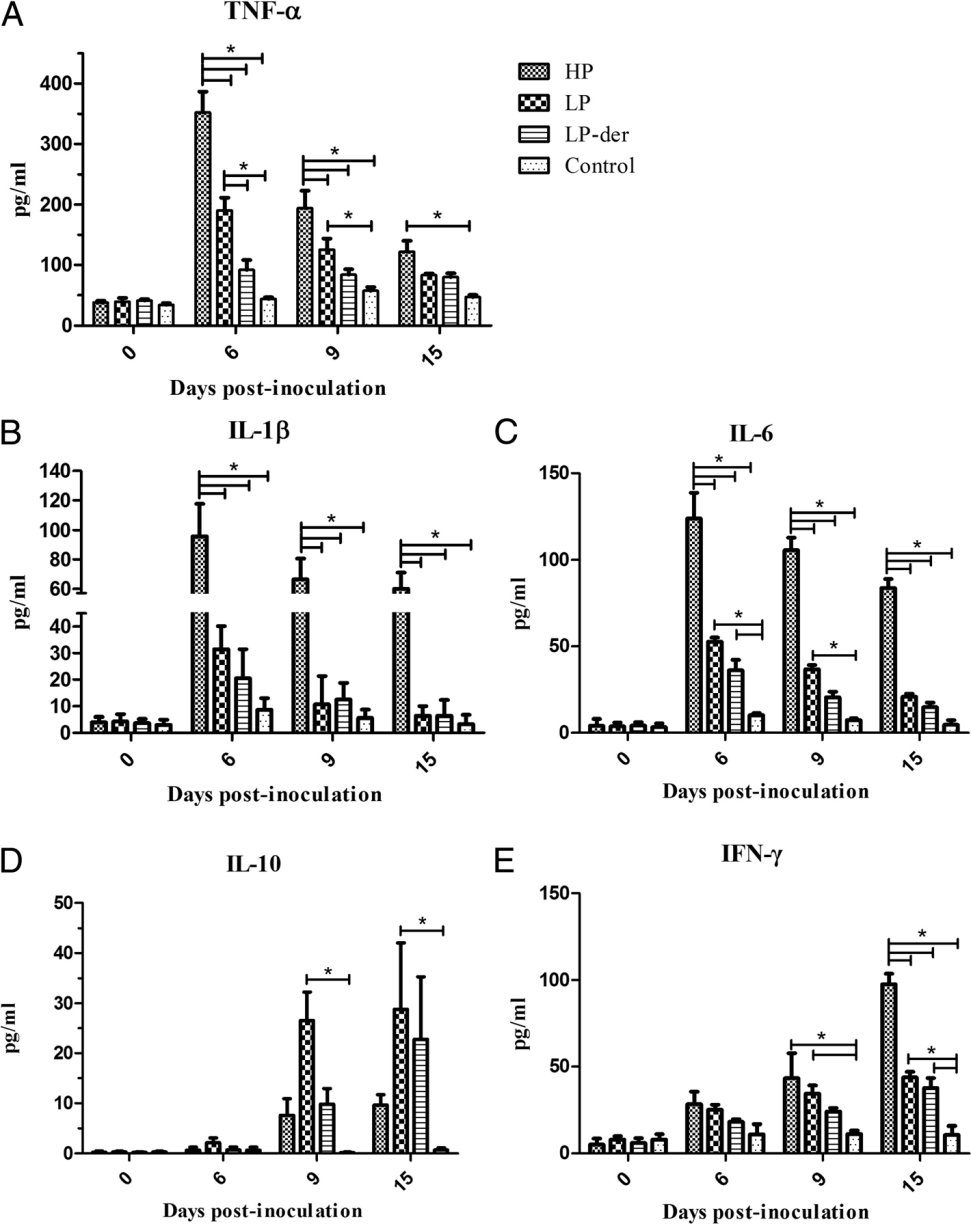
**Serum levels of porcine TNF-α (A), IL-1β (B), IL-6 (C), IL-10 (D), and IFN-γ (E) were measured with commercial ELISA kits.** Data are represented as mean (± SEM) generated from 6 pigs in all groups at 0 to 15 d.p.i. and 4 pigs in group HP at 9 to 15 d.p.i. (*) indicates a significant difference (*p* < 0.05) between groups.

At 0, 6, 9, and 15 d.p.i., blood samples were collected and the serum levels of TNF-α, IL-1β, IL-10, IL-6, and IFN-γ were detected. As shown in Figure 4, the levels of TNF-α, IL-1β, and IL-6 in the three viral groups rapidly increased at 6 d.p.i. and then gradually decreased until 15 d.p.i. In group HP, cytokine levels were significantly higher (*p* < 0.05) than those in groups LP and LP-der. Meanwhile, LP-PRRSV-inoculated pigs had relatively higher levels of TNF-α, IL-1β, and IL-6 compared to those in group LP-der (*p* < 0.05). In the LP-der and control groups, there were no significant changes in those cytokine levels during the experiment (Figure 4).

Interestingly, IL-10 levels in group HP were lower than those in group LP at 9 and 15 d.p.i., even though they were increased in group HP at all time points compared with those in the control group. Meanwhile, IL-10 levels in group LP-der were lower than those in group LP, but higher than those in the control group (Figure 4D).

Although low levels of serum IFN-γ were detected individually in PRRSV-infected pigs at 6 and 9 d.p.i., the levels in group HP pigs were significantly higher than those in the other groups at 15 d.p.i. (*p* < 0.05).

#### TLR mRNA expression levels in PAMs and cerebral medulla

**Figure 5:**
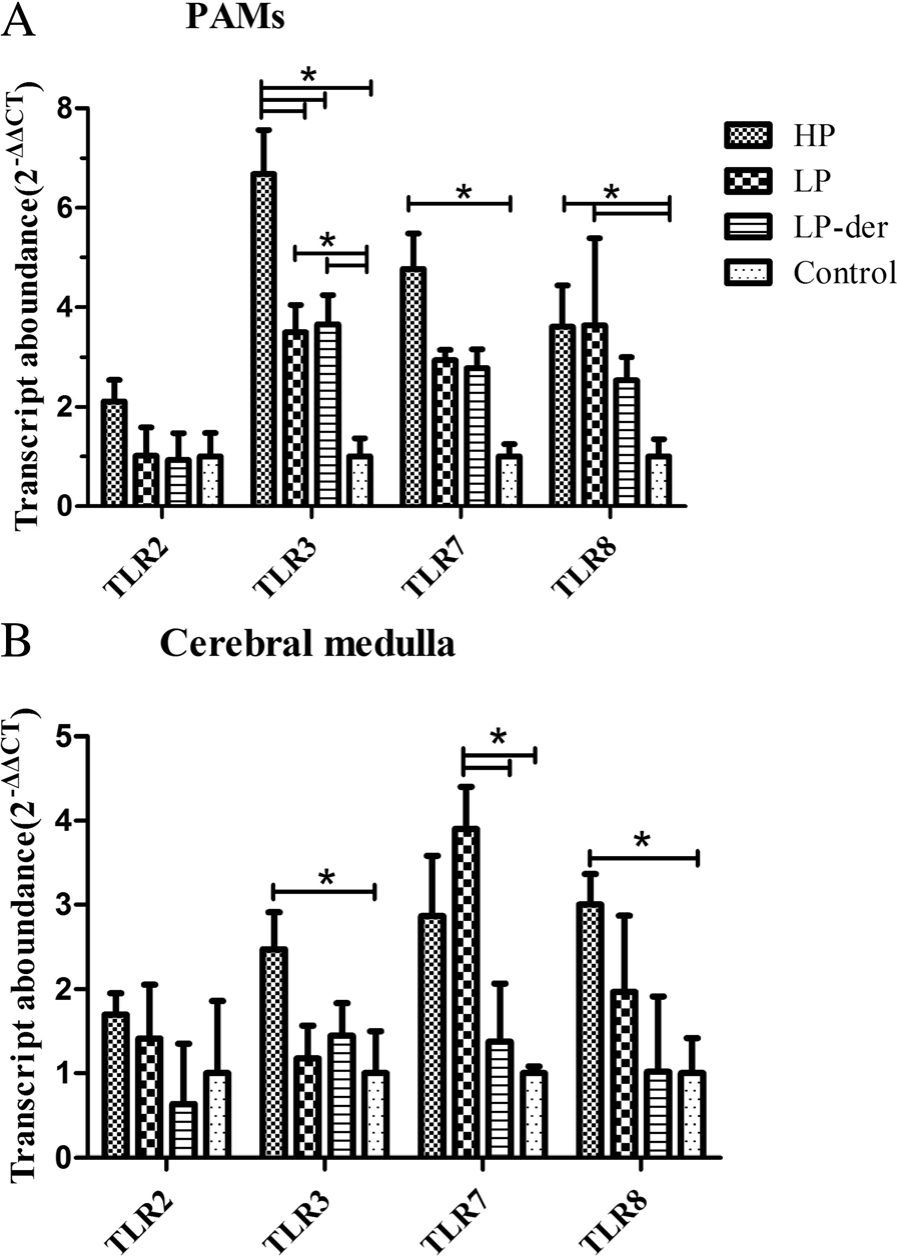
**TLR mRNA expression in porcine PAMs (A) and cerebral medullar tissues (B) determined by real-time PCR.** PAMs and cerebral medullar tissues were collected at necropsy conducted at 15 d.p.i. (*) indicates a significant difference (*p* < 0.05) between groups. Data are presented as means (± SEM) generated from 6 pigs in the LP, LP-der, and control groups or 4 pigs in group HP.

The expression levels of TLR2, 3, 7, and 8 mRNA were examined individually by real-time polymerase chain reaction (PCR) in porcine alveolar macrophages (PAMs) and the cerebral medulla of pigs infected with the different PRRSV isolates. As shown in Figure 5A, the TLR3, 7, and 8 mRNA levels in PAMs of all PRRSV-infected pigs were higher than those of mock-infected pigs and the levels of TLR3 mRNA in PAMs from group HP-PRRSV were highest among all groups (*p* < 0.05). Meanwhile, the TLR2 mRNA expression level in group HP PAMs was also relatively higher than those of the other three groups (*p* > 0.05).

In the cerebral medulla, TLR3, 7, and 8 mRNA expression levels were significantly increased after HP-PRRSV challenge compared to those in group LP-der and mock-infected pigs (*p* < 0.05). TLR7 expression in group LP pigs was significantly higher than those in the LP-der (*p* < 0.05) and mock-infected groups (*p* < 0.01). However, there were no significant differences in TLR3, 7, and 8 mRNA levels in group LP-der compared with those of the control pigs (Figure 5B).

### Cytokine expression in PAMs and peripheral blood mononuclear cells (PBMCs) after PAMP stimulation

**Figure 6:**
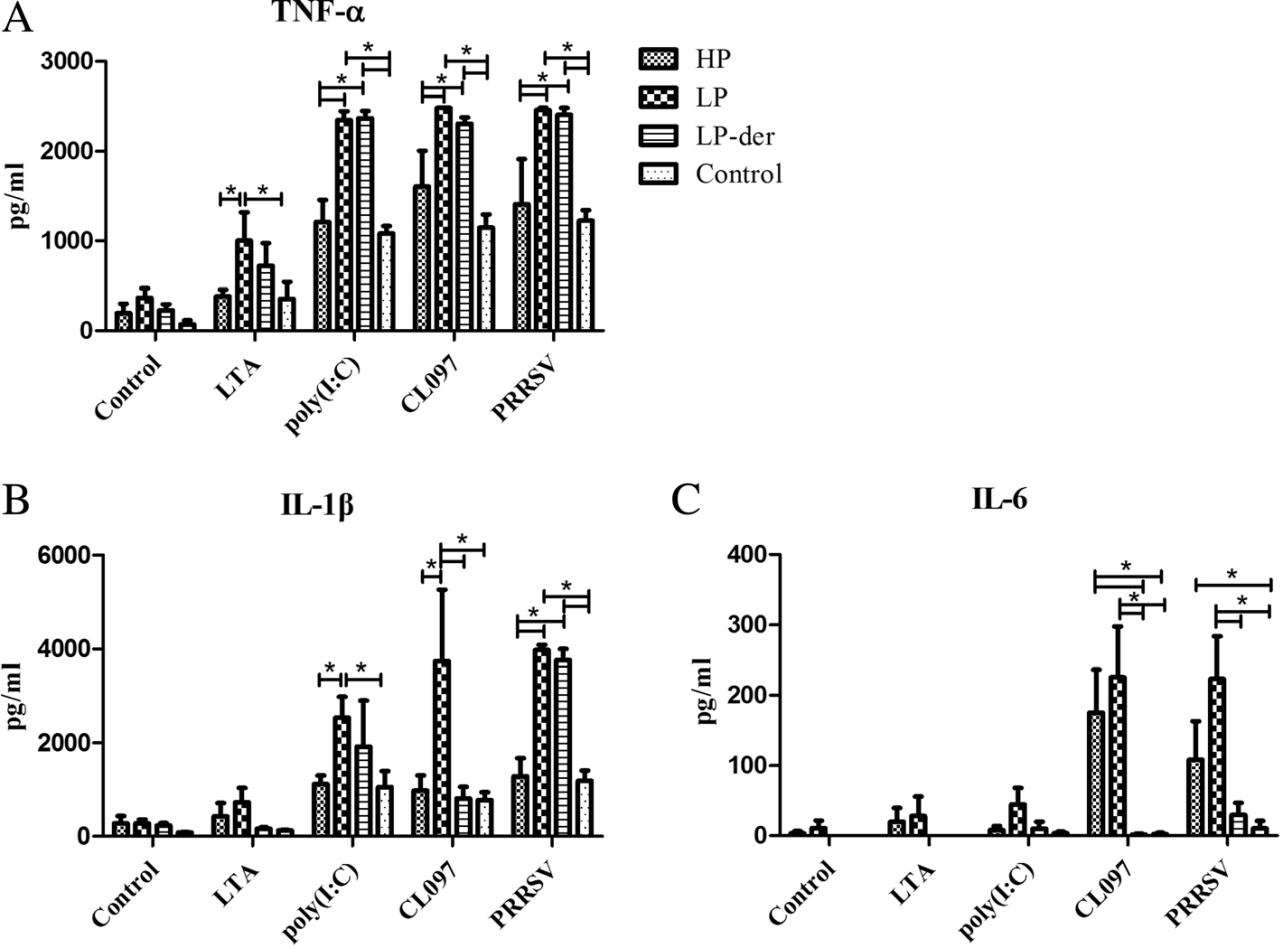
**Cytokine production in PAMs following stimulation with LTA, poly(I:C), CL097, or PRRSV from HP, LP, and LP-der PRRSV-infected and mock-infected groups.** Cytokine concentrations in the supernatants of stimulated PAMs were determined by ELISA. Data are presented as means (± SEM) generated from 6 pigs in the LP, LP-der, and control groups or 4 pigs in group HP. (*) indicates a significant difference between groups (*p* < 0.05).

PBMCs and PAMs were collected from PRRSV- and mock-infected pigs and individually treated with lipoteichoic acid (LTA), poly(I:C), CL097, and PRRSV, or they remained untreated as controls. The culture supernatants were then collected to determine cytokine concentrations using an enzyme-linked immunosorbent assay (ELISA). As shown in Figure 6, TNF-α levels in PAMs from all groups were significantly increased after being stimulated individually with poly(I:C), CL097, or PRRSV (*p* < 0.05) compared with unstimulated PAMs. The TNF-α concentrations in poly(I:C)-, CL097-, and PRRSV-treated PAMs from LP- and LP-der-PRRSV-infected pigs were significantly higher than those in HP- and mock-infected pigs (*p* < 0.05) (Figure 6A). Notably, CL097 and poly(I:C) significantly up-regulated IL-1β expression in PAMs from group LP compared to those from HP- and mock-infected pigs (*p* < 0.05) (Figure 6B). IL-6 levels were significantly increased in CL097- and PRRSV-treated PAMs from groups HP and LP compared to those in group LP-der and the controls (*p* < 0.05) (Figure 6C).

**Figure 7:**
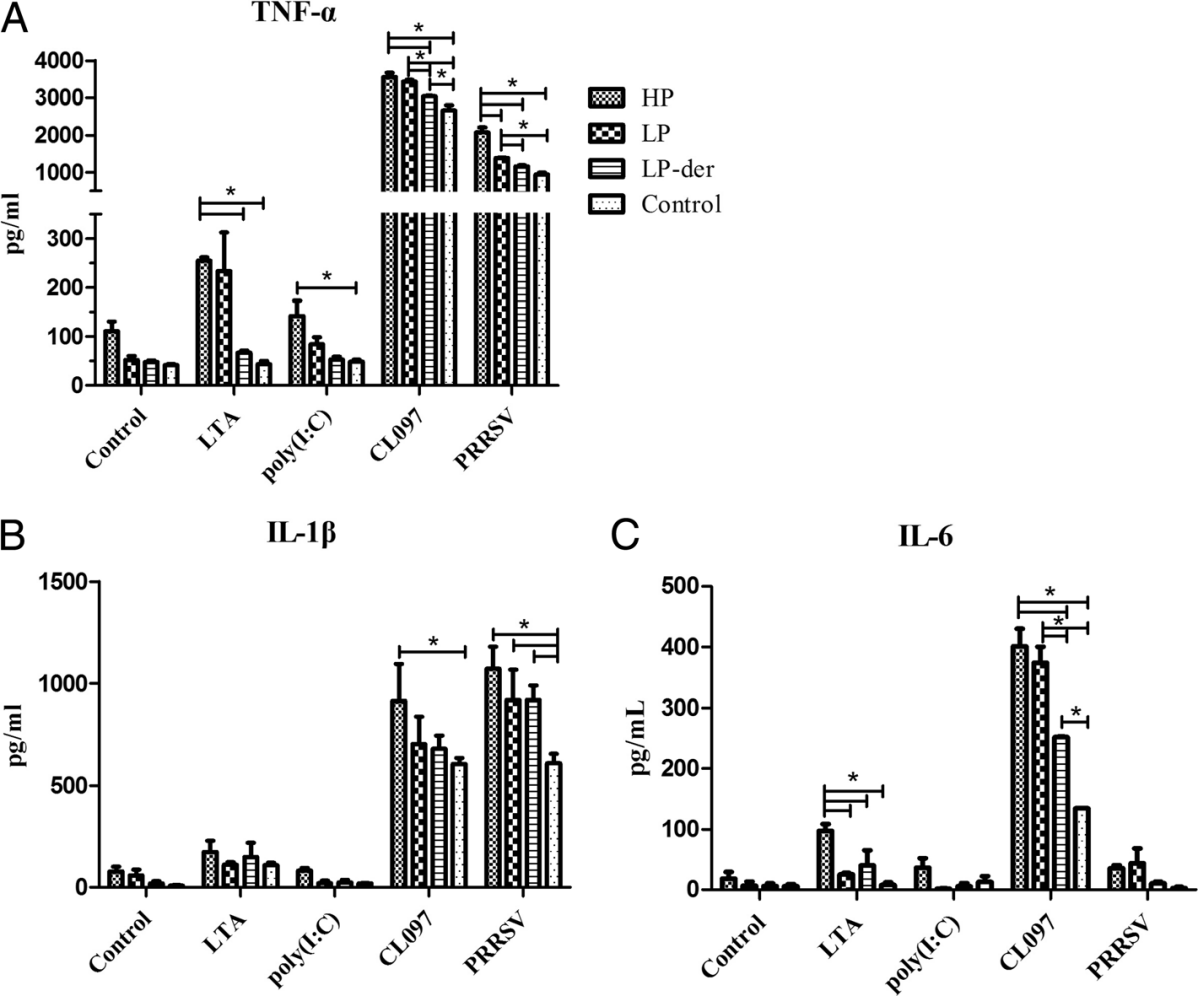
**Cytokine production in PBMCs following stimulation with LTA, poly(I:C), CL097, or PRRSV from PRRSV- and mock-infected pigs.** Cytokine concentrations in the supernatants of stimulated PBMCs were determined by ELISA. Data are presented as means (± SEM) generated from 6 pigs in the LP, LP-der, and control groups or 4 pigs in group HP. (*) indicates a significant difference between groups (*p* < 0.05).

Meanwhile, the TNF-α, IL-1β, and IL-6 levels in CL097-stimulated PBMCs from all groups were significantly higher than those in the untreated cells from the corresponding groups (*p* < 0.05) and their levels in CL097-treated PBMCs from groups HP, LP, and LP-der were significantly higher than those from mock-infected groups (*p* < 0.05). The TNF-α and IL-6 concentrations in CL097-stimulated PBMCs from group HP were significantly higher than those from group LP-der (*p* < 0.05). TNF-α secretion in poly(I:C)- and LTA-stimulated, and IL-6 in LTA-stimulated PBMCs from group HP were statistically higher than those from group LP-der (*p* < 0.05). In addition, TNF-α and IL-1β levels in PRRSV-treated cells from groups HP, LP, and LP-der were significantly higher than those in the mock-infected group (*p* < 0.05) (Figure 7).

## Discussion

Pro-inflammatory cytokines, such as IL-1, IL-6, IFN-γ, and TNF-α, are among the first cytokines produced by alveolar macrophages in response to viral infections. The increased circulation levels of these cytokines are responsible for the symptoms of acute systemic inflammation, including fever, depression, and anorexia, which have been associated with cytokine production in lung tissues of PRRSV-infected animals [16,17]. In the present study, HP-PRRSV-infected pigs showed clear clinical signs and high mortality rates and had severe interstitial pneumonia and pneumocytic hyperplasia in their lung tissues. HP virulence was markedly greater than that of LP, which was isolated from clinically ill piglets, and did not have the 30-aa deletion in NSP2 compared to that of PRRSV strain VR-2332. These results were in agreement with earlier in vivo experiments, in which pigs were infected with highly pathogenic American-type PRRSV [4,18]. The NT0801-F70 strain derived from NT0801 by propagation in MARC-145 cells to 70 passages showed almost no pathogenicity in piglets. BB0907-infected pigs showed more severe clinical signs and had significantly higher serum levels of TNF-α, IL-1β, and IL-6 than those infected with NT0801. Therefore, PRRSV BB0907 and NT0801 isolates could be termed as HP-PRRSV and LP-PRRSV, respectively. IL-1, IL-6, IFN-γ, and TNF-α expression levels were correlated to PRRSV virulence, which was similar to the results of other reports regarding American serotype 2 HP-PRRSV strain rJXwn06 rescued from an infectious clone of Chinese HP-PRRSV, and VR2332 [19], virulent European subtype 3 strain Lena, and low virulent subtype 1 strains, Belgium A and LV [19]. Because the clinical signs in pigs infected with HP- and LP-PRRSV gradually disappeared during the period of 10 to 13 d.p.i, all pigs were euthanized for pathological detection at 15 d.p.i., which was a shorter period than that used in another report [18].

TLRs 3 and 7/8 recognize dsRNA and ssRNA, respectively, and play key roles in viral-mediated innate immune responses. The expression of most TLRs, including TLR3, 7, and 8, were up-regulated in monocytes of chronic hepatitis C virus–infected patients [19]. Reportedly, TLR3 and 7 mRNA were up-regulated in peripheral and brain tissues of PRRSV-infected pigs. The up-regulation of TLRs, as well as the corresponding neuroinflammatory response, was likely due to the presence of PRRSV in the brain and cytokine-based signals from the peripheral immune system [20]. Infection of pigs with PRRSV tended to up-regulate the mRNA expression of TLR2, 3, 4, 7, and 8 in at least one of the lymphoid tissues and PAMs [21]. In contrast, the down-regulation of TLR7 and 8 by poly(I:C) stimulation and PRRSV infection was observed in both PAMs and immature dendritic cells [22]. Interestingly, in the present study, we found that HP infection induced higher expression levels of TLR3, 7, and 8 mRNA in PAMs and cerebral medullar tissues than LP, indicating that the TLR expression levels were correlated with PRRSV virulence. Increased TLR expression is an ominous prognostic factor in the host, whereas low TLR expression may protect the host against excessive inflammation and tissue damage. Thus, differential TLR expression in PRRSV-infected pigs may contribute to infection susceptibility and underlying disease progression; however, this supposition remains to be further validated in future studies.

The TLR family can detect distinct microbial PAMPs and trigger the activation of specific signaling pathways leading to the transcription of inflammatory and anti-inflammatory cytokines [8]. The exaggerated production of proinflammatory cytokines via the activation of the TLR pathway might contribute to the exacerbated clinical symptoms in combined infections of PRRSV and secondary pathogens. However, the exact mechanisms of interaction between PRRSV and secondary bacterial/viral pathogens remain unclear. Recognition of pathogens requires TLR-mediated signals to initiate innate and subsequent adaptive immune responses. Conceivably, modulated TLR expression following PRRSV infection in pigs, along with specific PAMPs presented by the secondary pathogens, can significantly influence disease outcome of the combined infection [23]. An in vitro model was established to investigate pro-inflammatory cytokine production by macrophages in response to inoculation with PRRSV and lipopolysaccharides [23]. Synthetic RNA compounds, such as poly(I:C), can activate TLR3-expressing cells [24] and the water-soluble derivative of the imidazoquinoline compound CL097, which acts an a TLR7/8 ligand in the induction of NF-κB activation [25]. In the present study, our results showed that TNF-α levels in poly(I:C)-stimulated PBMCs from group HP were significantly increased compared to those from the control group. TNF-α and IL-6 levels in CL097-stimulated PBMCs from groups HP and LP were statistically greater than those from group LP-der. However, the levels of TNF-α, IL-1β, and IL-6 in PAMs from group HP were significantly lower than those from group LP. These results might be explained by the low activity of PAMs with high PRRSV load from group HP. Analysis of in vitro PRRSV infectivity in PAMs indicated that pre-treatment with the TLR3 ligand, dsRNA, suppressed PRRSV infection (Miller et al., 2009). The authors suggested that TLR3 activation might stimulate protective activity against PRRSV infection. Thus, the up-regulation of TLR3 and TLR7 in lung and brain tissues of PRRSV-infected pigs may restrict viral replication.

IL-10 is an immunomodulatory cytokine that is able to inhibit the synthesis and release of other cytokines [26], thereby inhibiting cell-mediated immunity and extending the duration of viremia in the peripheral blood during the early stage of infection [27]. Although, IL-10 expression was found to be significantly correlated with PRRSV replication [17], it has been reported that some PRRSV strains induce IL-10 secretion in infected macrophages while others do not [28,29]. In this study, HP-infected pigs developed low serum IL-10 levels by 15 d.p.i and the level of viremia in the peripheral blood was significantly higher than that in the negative control group. Meanwhile, LP-infected pigs developed higher IL-10 levels compared to those in the other groups. The vFL12 strain, a highly pathogenic PRRSV strain derived from an infectious clone, did not up-regulate IL-10 at the mRNA or protein levels in either infected macrophages or dendritic cells in vitro or in vivo [28,29], suggesting that IL-10 release might not be a part of the PRRSV virulence mechanism.

Usually, HP-PRRSV replicates more efficiently than LP PRRSV and displays an expanded tissue tropism in vivo [6,30,31]. For example, infection with the virulent European subtype 3 strain Lena resulted in higher serum viral titers compared to low virulent subtype 1 strains, such as Belgium A and LV [6,30,31]. HP-PRRSV strain HuN4 caused higher viral loads in peripheral blood samples than its derivative strain HuN4-F112 (obtained by propagation in MARC145 cells for 112 passages) [6,30,31]. Here, our results showed that LP-PRRSV strain NT0801 induced greater viremia in pigs than its derivative strain NT0801-F70 and those infected with HP-PRRSV strain BB0907 had milder PRRSV viremia than that due to LP-PRRSV strain NT0801, which was isolated from a piglet herd that experienced a morbidity of ~40% and mortality of ~20%. Strain NT0801 shares a homology of 96.7% with the HP-PRRSV SY0608 isolate (GenBank accession no.: EU144079), but does not have the 30-aa deletion in NSP2. Furthermore, no obvious recombination signal was observed by comparing its sequence with other PRRSV isolates with different virulence (data not shown). However, the mechanism behind the high viremia level induced by LP-PRRSV strain NT0801 requires further exploration in future studies.

In addition, PRRSV reportedly replicates only in macrophages, but not in monocytes or other peripheral blood cells [32]. However, in the present study, when PBMCs were isolated from pigs infected with the respective PRRSV strain, cultured, and treated with TLR ligands or infected with PRRSV, we found that PRRSV also induced a strong effect on TNF-α and IL-1β secretion, similar to that of the TLR-ligand CL097, although PBMCs are not supposed to contain macrophages. This result may be explained by the maturity of the PBMCs or the activation of the PRRSV antigen protein [32].

## Conclusions

Here, we found that HP was a stronger inducer of TLR 3, 7, 8 expression and IL-1β, IL-6, TNF-α, IFN-γ production compared to LP and LP-der. Furthermore, HP enhanced production of TNF-α, IL-1β, and IL-6 in PBMCs following stimulation with CL097 more so than LP or LP-der, whereas LP enhanced TNF-α and IL-1β secretion in poly(I:C)- and CL097-stimulated PAMs from PRRSV-infected pigs. Together, our results indicate that PRRSV strains with different virulence can differently modulate the immune responses in swine and should be helpful in the development of more efficacious vaccines against PRRSV infection.

## Materials and methods

### Viruses

A highly pathogenic PRRSV (HP-PRRSV) isolate BB0907 (propagation in MARC-145 cells for 6 passages) (HP) (GenBank no. HQ315835) was isolated in Guangxi Province, China, in 2009. The low pathogenic PRRSV (LP-PRRSV) isolate NT0801 (LP) (GenBank no. HQ315836) was isolated in Jiangsu Province, China, in 2008, and its derivative strain NT0801-F70 (LP-der) was obtained after propagated in MARC-145 cells to 70 passages. The titers of the viral stocks were determined by cytopathic effect (CPE) in MARC-145 cells. The titers of BB0907, NT0801, and NT0801-F70 viral stocks were 10^5.87^, 10^6.25^, and 10^6.5^ TCID_50_/ml, respectively.

### Experimental design

Twenty-four, 6-week-old, healthy, crossbred piglets free of PRRSV and porcine circovrius type 2 (PCV2) infection were randomly divided into four groups with 6 pigs per group. The pigs in group HP, LP, and LP-der were intranasally inoculated with 2 mL of the BB0907, NT0801, and NT0801-F70 strains of PRRSV, respectively, with 3 × 10^5.5^ TCID_50_/mL per pig. The pigs in the control group were intranasally inoculated with 2 mL of Dulbecco modified Eagle’s medium (DMEM). The animals were monitored for 15 days post inoculation (d.p.i.). Rectal temperatures and clinical signs were observed daily. At 0, 3, 6, 9, 12, and 15 d.p.i., the blood and sera samples were collected for the detection of PRRSV, IL-1β, IL-6, IL-10, TNF-α, and IFN-γ. At the end of the experiment, all pigs were euthanized for necropsy. Peripheral blood mononuclear cells (PBMCs), porcine alveolar macrophages (PAMs), and lung and brain tissues were collected for further examination.

This study was approved by the Animal Care and Ethics Committee of Nanjing Agricultural University (permit number: IACECNAU20111105). All authors complied with the “Animal Research: Reporting In Vivo Experiments” (ARRIVE) guidelines [33].

### Clinical and pathology examination

The clinical conditions of the pigs were evaluated daily after challenge as previously reported [34]. Briefly, scores ranged from 1–4, reflecting the severity of the illness, were determined by sum of daily observations of behavior, respiration, and cough. Gross lung lesions were evaluated at necropsy. Gross lesions of each lobe were scored and estimated as the percentage of lung with grossly visible pneumonia, and the overall level of gross lung pathology was determined. Then, the lung tissues were histologically evaluated as previously reported [35].

### Preparations of PBMCs and PAMs

Blood samples from pigs were collected from the jugular veins into blood collection tubes containing heparin lithium before the necropsies. PBMCs were isolated by density gradient centrifugation using lydroxypropylmethyl cellulose (TBDscience, Tianjing, China). Cells were washed three times in phosphate-buffered saline (PBS), counted, and resuspended in RPMI-1640 medium (Gibco-Invitrogen, Carlsbad, CA, USA)). PBMCs were dispensed into 24-well-plates (Corning, Inc., Corning, NY, USA) at 10^6^ cells/well in a volume of 500 μL. The cells were stained and subjected to differential counting based on cell morphology. For PBMC cultures, both adherent and non-adherent cells were used for the study.

The PAMs were isolated from bronchoalveolar lavage by centrifugation as described previously [21]. PAMs were diluted to 2 × 10^6^ cells/mL and dispensed into 24-well plates (Corning, Inc.) or 25-cm^2^ flasks (Corning, Inc.) in a volume of 500 μL or 5 mL. The differential counting was performed on stained cytospin slides of the PAMs. Non-adherent cells were removed by gentle aspiration of the culture supernatant and replaced with fresh media.

### Stimulation of PAMs and PBMCs with PRRSV or microbial PAMPs

PAMs and PBMCs dispensed into 24-well plates were treated with LTA from *Staphylococcus aureus* (10 μg/mL; InvivoGen, San Diego, CA, USA), poly(I:C) (10 μg/mL; InvivoGen), CL097 (5 μg/mL; InvivoGen), PRRSV (BB0907, NT0801, NT0801-F70, 0.1MOI), or they remained untreated (control). Stimulated PAMs and PBMCs were then cultured in a 5% CO_2_ incubator at 37°C for 48 h. Supernatants were then collected from the cell cultures for cytokine concentration determination by ELISA. To investigate TLR mRNA expression, PAMs in 25-cm^2^ flasks obtained from PRRSV- and mock-infected pigs were used for RNA extraction.

### Cytokine measurement

TNF-α, IL-1β, IL-6, IL-10, and IFN-γ were measured in pig serum or the culture supernatants using porcine specific ELISA kits (R&D Systems, Minneapolis, MN, USA) according to the manufacture’s instructions. A standard curve was generated using known concnetrations. All detections were performed in parallel.

### Real-time PCR for detection of PRRSV in serum

Total RNA was extracted from serum using TRIzol reagent (Invitrogen, Carlsbad, CA, USA) according to the manufacturer’s instructions. Reverse transcription was performed using M-MLV Reverse Transcriptase (Promega, Madison, WI, USA) in a 25-μL reaction mixture containing 2 μg of RNA according to the manufacturer’s instructions. Two microliters of the RT reaction mixture was subjected to real-time PCR analysis using ORF7-specific primers (sense: 5’-AAT AAC AAC GGC AAG CAG CAG-3’; antisense: 5’-CCT CTG GAC TGG TTT TGC TGA-3’), and SYBR Green Real-time PCR Master Mix (Toyobo Co., Ltd., Osaka, Japan), according to the Manufacturer’s recommendations. The reaction procedure included one a denaturing stage at 95°C for 2 min, followed by 40 cycles at 95°C for 15 s and 60°C for 1 min. To confirm specific amplification, melting curve analysis of the RT-PCR products was performed according to the manufacturer’s protocol. Real-time PCR was performed in an ABI PRISM 7300 sequence detection system and analyzed with ABI PRISM 7300_SDS_ software (Applied Biosystems, Foster City, CA, USA). For each assay a standard curve was generated using serially diluted PRRSV as described previously [36]. Slope = −3.551481; R^2^ = 0.988176; PCR efficiency = 0.9123.

### Real-time PCR for detection of TLR mRNA

Total RNA was extracted from PAMs and brain tissues as described above. Based on the sequence of porcine TLR2, TLR3, TLR7, TLR8, β-actin, and 18S ribosomal RNA (18S rRNA) (GenBank number in Table 1), the probes and primers were designed using Primer Express version 3.0 software (Applied Biosystems) as shown in Table 2. All Taqman minor groove binding oligonucleotide probes contained FAM as a fluorescent reporter dye at the 5’ end and TAMRA as a quencher dye at the 3’ end. Appropriate reference genes for real-time RT-PCR were selected based on preliminary analyses indicating similar expression of the control genes among the four treatment groups. β-actin (PAMs) and 18S rRNA (brain) were used as reference genes for data normalization. The levels of TLRs mRNA from cDNA in the samples were calculated using the following formula: 2^-ΔΔCt^ = 2^ΔCt TLRs^ / 2^ΔCt β-actin^. ΔCt TLRs = Ct TLRs of the cells at indicated time-Ct TLRs of the cells at corresponding time; ΔCt β-actin = Ct β-actin of the cells at corresponding time -Ct β-actin of the cells at corresponding time [20].

**Table 2:** Sequences of porcine-specific real-time RT-PCR primers and TaqMan probes

**Genes**^**a**^	**Primer sequence (5’-3’)**^**b**^	**Probe sequence (5’-3’)**^**c**^	**Accession number**^**d**^
TLR2	F:TGCTGGAACCCATCGAGAA	AAGGCCATTCCCCAGCGTTTCTGTAA	GU138028
	R:AGGTAGGTCCTGGTGTTCATTATCTT		
TLR3	F:CCCAGTGATTCTTTTTGATACATCA	CCTGCAAAGACAGTGCCCCATTTGA	GU013759
	R:CATACTGGCACTTATCATGAAAAAGAG		
TLR7	F:CCAACAACCGGCTTGATTTAC	CAACAGCATTCGAAGAGCTACGCAACC	GU013760
	R:TCTGATTGAAAATAGTGGCTGTTACTACT		
TLR8	F: AAAAAGCACGTCCCTGAAAGAA	TTTTCAGTGGAAACCGCCTGGACCTT	GU013761
	R:TACCTGTCATCTTGGGCATTCC		
β-actin	F:TCTTCCAGCCCTCCTTCCT	AGTCCTGCGGCATCCACGAGACC	U07786
	R:ACGTCGCACTTCATGATCGA		
18S rRNA	F:CCCCAACTTCTTAGAGGGACAA	TGGCGTTCAGCCACCCGAGATTT	DQ437859
	R:GGGCATCACAGACCTGTTATTG		

^a^TLR2, Toll-like receptor 2; TLR3, Toll-like receptor 3; TLR7, Toll-like receptor 7; TLR8, Toll-like receptor 8; 18S rRNA, 18S ribosomal RNA.

^b^F, forward primer; R, reverse primer.

^c^All probes contained FAM as a fluorescent reporter dye at 5’end and TAMRA as quencher dye at the 3’end.

The total PCR reaction volume was 20 μL, including 2 μL of cDNA templates, 10 μM of each primer and probe, 2 × Premix Ex Taq (Takara Co., Ltd., Shinga, Japan). The PCR conditions were as follows: predenaturing at 95°C for 30 s and 45 cycles of denaturing at 95°C for 15 s, annealing at 56°C for 15 s, and an extension at 72°C for 31 s. The information of the real-time PCR results was shown in Additional file 1: Figure S1. Those PCR efficiencies were 0.91068 ~ 1.08137.

### Statistical analysis

Results were analyzed for significance with the two-way analysis of variance test using GraphPad PRISM software (version 5.02 for Windows; http://www.graphpad.com/). A *p*-value < 0.05 was considered significantly significant.

## Abbreviations

HP: Highly pathogenic PRRSV strain BB0907; HP-PRRSV: Highly pathogenic PRRSV; LP: Low pathogenic PRRSV strain NT0801; LP-der: Low pathogenic PRRSV derivative strain NT0801-F70; LP-PRRSV: Low pathogenic PRRSV; PAMs: Porcine alveolar macrophages; PBMCs: Peripheral blood mononuclear cells; PRRSV: Porcine reproductive and respiratory syndrome virus; TLRs: Toll-like receptors.

## Competing interests

The authors declare that they have no competing interests.

## Author contributions

LZ performed most of the experiments and wrote the manuscript. PJ critically revised the manuscript and the experimental design. JL collected samples and gathered clinical data. JB and XW analyzed the animal experimental data and edited the draft manuscript. YL analyzed the histopathological data. All authors read and approved the final version of the manuscript.

## Supplementary Material

Additional file 1: Figure S1 Standard curves for quantitation of TLRs, β-actin and 18S rRNA by TaqMan Real-time PCR. (A) Amplification curves obtained with serial dilutions of TLRs, β-actin and 18S rRNA genes. The *x*-axis shows the number of PCR cycles and the *y*-axis shows the normalized fluorescence intensity (Rn). (B) The standard curves of TLRs, β-actin and 18S rRNA genes show comparable slopes, indicating similar PCR efficiency. Click here for file
