# Supplementary figures and images for: Comparative expression of Toll-like receptors and inflammatory cytokines in pigs infected with different virulent porcine reproductive and respiratory syndrome virus isolates
Source: Virol J. 2013 Apr 30;10:135. doi: 10.1186/1743-422X-10-135 (PMC3673858; doi:10.1186/1743-422X-10-135)

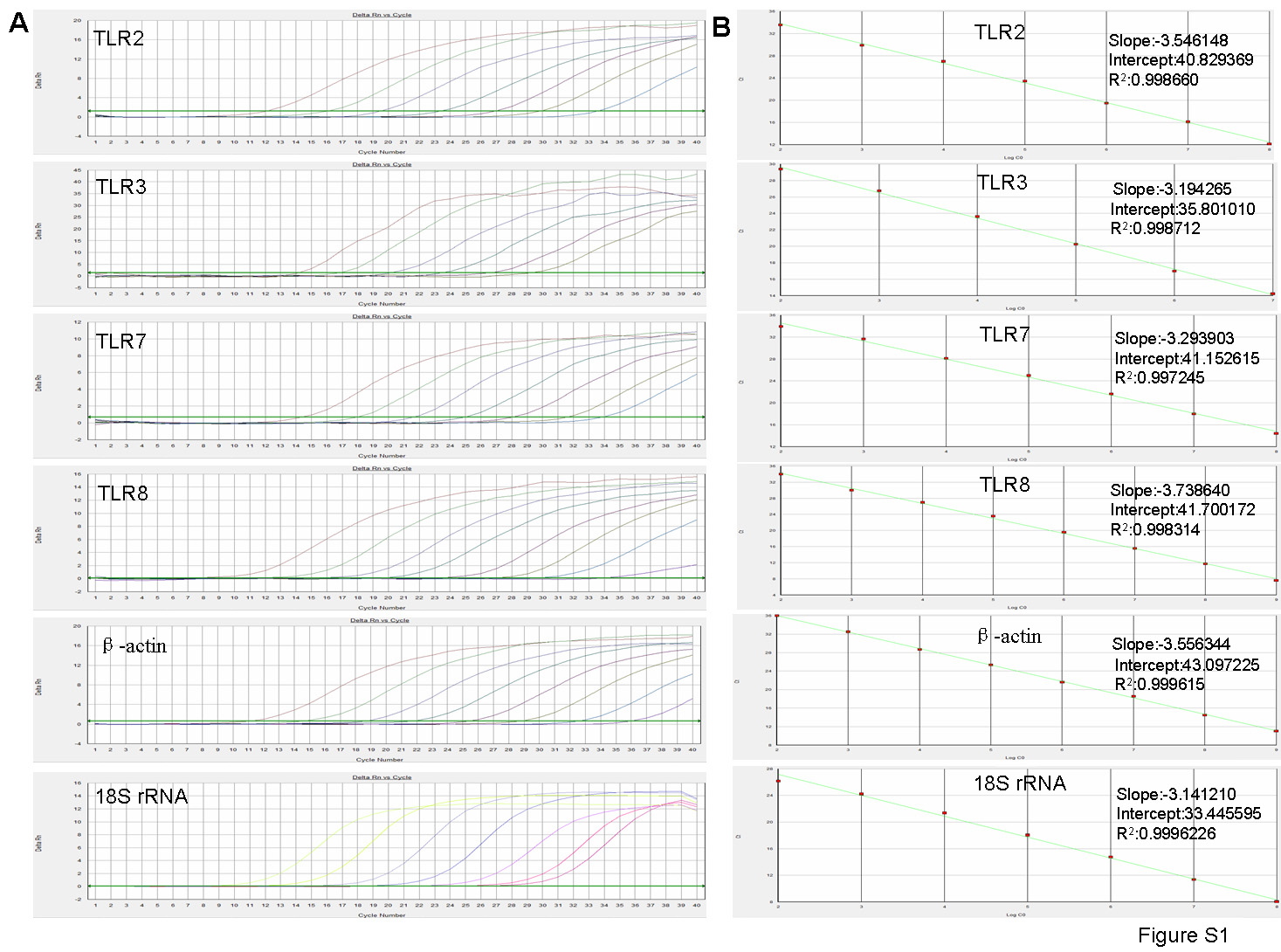

Supplement: Additional file 1: Figure S1 — Standard curves for quantitation of TLRs, β-actin and 18S rRNA by TaqMan Real-time PCR. (A) Amplification curves obtained with serial dilutions of TLRs, β-actin and 18S rRNA genes. The x-axis shows the number of PCR cycles and the y-axis shows the normalized fluorescence intensity (Rn). (B) The standard curves of TLRs, β-actin and 18S rRNA genes show comparable slopes, indicating similar PCR efficiency. [file 1743-422X-10-135-S1.jpeg]
